# Dynamic Perturbations of the T-Cell Receptor Repertoire in Chronic HIV Infection and following Antiretroviral Therapy

**DOI:** 10.3389/fimmu.2015.00644

**Published:** 2016-01-11

**Authors:** James M. Heather, Katharine Best, Theres Oakes, Eleanor R. Gray, Jennifer K. Roe, Niclas Thomas, Nir Friedman, Mahdad Noursadeghi, Benjamin Chain

**Affiliations:** ^1^Division of Infection and Immunity, University College London, London, UK; ^2^Centre for Mathematics and Physics in the Life Sciences and Experimental Biology (CoMPLEX), University College London, London, UK; ^3^Department of Immunology, Weizmann Institute, Rehovot, Israel

**Keywords:** T-cell, TCR, repertoire, sequencing, HIV, AIDS, ART

## Abstract

HIV infection profoundly affects many parameters of the immune system and ultimately leads to AIDS, yet which factors are most important for determining resistance, pathology, and response to antiretroviral treatment – and how best to monitor them – remain unclear. We develop a quantitative high-throughput sequencing pipeline to characterize the TCR repertoires of HIV-infected individuals before and after antiretroviral therapy, working from small, unfractionated samples of peripheral blood. This reveals the TCR repertoires of HIV^+^ individuals to be highly perturbed, with considerably reduced diversity as a small proportion of sequences are highly overrepresented. HIV also causes specific qualitative changes to the repertoire including an altered distribution of V gene usage, depletion of public TCR sequences, and disruption of TCR networks. Short-term antiretroviral therapy has little impact on most of the global damage to repertoire structure, but is accompanied by rapid changes in the abundance of many individual TCR sequences, decreases in abundance of the most common sequences, and decreases in the majority of HIV-associated CDR3 sequences. Thus, high-throughput repertoire sequencing of small blood samples that are easy to take, store, and process can shed light on various aspects of the T-cell immune compartment and stands to offer insights into patient stratification and immune reconstitution.

## 1 Introduction

During the chronic phase of HIV infection, viral load and total T-cell numbers remain relatively stable. However, analysis of T-cell turnover reveals a more dynamic picture, with continuous virus turnover and rapid CD4 cell death offset by increased T-cell proliferation (1). HIV infection typically leads to development of AIDS, although this can be prevented with combination antiretroviral therapy (ART). ART rapidly reduces viremia to very low or undetectable levels and is associated with a long-term increase in peripheral blood CD4 T-cell numbers and reduced risk of secondary infections (2). In this study, we examine the structure of the T-cell receptor (TCR) repertoire in chronically infected treatment-naïve individuals and monitor the changes that occur immediately after viral replication is blocked by ART.

HIV infection affects many features of the immune system, and it remains unclear which changes are ultimately responsible for different aspects of the pathology. HIV infection of CD4 T-cells leads to an increased rate of cell death by both direct virological and indirect immunological mechanisms (3, 4). HIV also drives CD8^+^ antigen-specific clonal expansion as well as unspecific cell activation and exhaustion (5, 6).

Many studies have investigated TCRs in HIV-infected individuals. Flow cytometry, DNA hybridization, and quantitative PCR have shown decreased expression of certain V genes (7–10). Spectratyping analysis has revealed a skewing of CDR3 length distributions in certain V families, indicating clonal expansions (11–13). A decrease in TCR diversity associated with HIV infection has also been measured using a number of experimental approaches (14–16), although such observations have typically been limited to either antigen- or subset-specific populations. The overall impact of viral cytotoxicity, viral antigenicity, and immune escape on the clonal structure of the T-cell population remain incompletely understood.

The introduction of high-throughput DNA sequencing allows analysis of the TCR repertoire at greater depth than previous techniques, with quantitative, nucleotide-level resolution (17, 18). Previous analysis of TCR repertoires from HIV patients often involved complex cell fractionation protocols, which pose a challenge in a routine clinical setting. However, we demonstrate that high-throughput analysis of TCR repertoire profiles from unseparated whole blood samples can reveal multiple levels of dysregulation in HIV patients. The diversity of the TCR repertoire is profoundly diminished during chronic infection, with expansion of large private clones driving the formation of highly idiosyncratic repertoires. HIV infection is also associated with skewing of V gene usage and disruption of the population structures that typify healthy repertoires. These perturbations remained largely unchanged after 3 months of ART, despite viral load being reduced to undetectable levels in most patients coincident with a significant increase in CD4 T-cell numbers. However, ART was accompanied by major changes at the sequence level, with many rapid, dramatic changes of frequency. Diverse immunological parameters and rapid restructuring events can therefore be captured from even small blood samples, which stands to offer new methods to monitor immune perturbation and reconstitution in patients suffering from infectious or immunodeficient conditions.

## 2 Materials and Methods

### 2.1 Study Design

This laboratory study aimed to test whether and how deep sequencing of TCR repertoires from HIV patients might produce results of interest to clinical and immunological investigations. Adult HIV^+^ ART-naïve patients due to start treatment were recruited, with the first sixteen eligible candidates being processed. Exclusion criteria included febrile or AIDS-related illness; tumors; coinfection with Hepatitis B or C; immunomodulatory therapy, recent vaccination, or breaks in treatment. 2.5 ml of peripheral blood was drawn into Tempus tubes, and RNA was extracted as per the manufacturer’s instructions (Life Technologies). Residual gDNA was removed using the TURBO-DNase kit, and globin mRNA was depleted using GLOBINclear (Life Technologies). RNA was likewise prepared from consenting adult healthy controls. Human participation in this study was approved by UK Research Ethics Committee. All subjects gave written informed consent in accordance with the Declaration of Helsinki.

### 2.2 Unbiased TCR Amplification and Sequencing

Our protocol amplifies all rearrangements (within a chain) with a single primer pair, thus avoiding primer bias (Figure S1 in Supplementary Material). Addition of molecular barcodes to each cDNA molecule allows further correction for PCR amplification and errors. Primer sequences are shown in Table S1 in the Supplementary Material.

RNA was reverse transcribed using oligonucleotides (*α*RC2 and *β*RC2) directed against each constant regions’ 5′. 500 ng of RNA was mixed with 1 μl of each RC2 primer (10 μM) and 1 μl of a 10-mM dNTP mix and heated at 65°C, 5 min before rapid cooling on ice. 1 μl of DTT (0.1 μM), 1 μl of RNasin (20–40 U/μl, Promega), 1 μl of SuperScript III reverse transcriptase (RT) (200 U/μl, Life Technologies) and 4 μl of 5× FS buffer were added, before incubation at 55°C, 20 min, then heat inactivated at 70°C, 15 min. RT reactions were purified and eluted into 10 μl of Tris-Cl using MinElute columns (Qiagen).

cDNA was ligated to a 5′ RACE adapter, incorporating a random hexamer and SP2, one of the two sequencing primer sites, in 30 μl: 1× T4 RNA ligase buffer; 20 U T4 RNA Ligase 1 (NEB); 0.33 mM dATP; 25% PEG 8000; 1 mM hexammine chloride; 0.1 mg/ml acetylated-BSA, 0.33 μM SP2-6N ligation oligonucleotide and 5 μl of purified cDNA. Ligations were incubated for 23 h at 16°C and heat inactivated for 10 min at 65°C. Products were diluted with 70 μl of water and purified using a 1:1 ratio of AMPure XP beads (Beckman Coulter), eluting in 30 μl of water.

Ligated DNA underwent second strand synthesis, priming off SP2. Final concentrations in 50 μl were 0.2 μM dNTPs, 0.5 μM SP2, and 1 U Phusion, in 1X HF buffer. Thermal conditions were 95°C, 3 min; a slow ramp to 80°C, 10 s; another slow ramp to 58°C, 45 s; and final extension for 5 min at 72°C. Slow ramping involves descending at 0.5°C/s to encourage optimal annealing at the highest temperature. Bead-purified samples were split in two for separate chain-specific third strand synthesis. Reaction conditions were the same as the second strand reaction, except the SP2 primer was replaced with either SP1-6N-Ix-*α*RC1 or SP1-6N-Ix-*β*RC1 (“x” being a multiplexing index). Bead-purified samples then underwent “PCR1” to add the P5 and P7 elements: 0.2 mM each dNTP, 0.5 μM of each primer (P5-SP1 and P7-X-SP2), and 1 U Phusion in 1X HF buffer (“X” being another index). Initial denaturation was at 95°C for 3 min, before ramping slowly to 69°C, 15 s, and extended at 72°C, 1 min, before 3 cycles of 98°C, 10 s to 72°C, 1 min. Final extension was at 72°C for 5 min. Purified products were then subjected to “PCR2,” to amplify to sufficient concentrations. Reaction conditions were the same as in PCR1, except primers used were P5s and P7. The PCR program began with initial denaturation at 95°C, 3 min, before 23 cycles of: 98°C, 10 s; 69°C, 15 s; 72°C, 40 s, then final extension at 72°C for 5 min and bead purification. Samples thus received 27 cycles of PCR in total. Final products were quantified on a Qubit (Life Technologies) and sized on a Bioanalyzer (Agilent) before dilution to 4 nM. Normalized libraries were sequenced on an Illumina MiSeq, using 2 × 250 PE kits.

### 2.3 Data Analysis

Demultiplexed FASTQ files were analyzed using a modified version of the TCR assignation software Decombinator, developed previously in our lab (19). This modified Decombinator additionally outputs barcode sequence information (comprised of the two random hexamer sequences added prior to amplification, concatenated together) in addition to the V, J, and insert information. This barcode information is then used to error-correct the TCR sequences: as the probability of any two identical TCRs acquiring the same 12-mer barcode (from the 4^12^ possibilities) is low, sequences that share a barcode and yet do not match the most frequent (within a 20% nucleotide identity threshold across the Decombinator-defined V–J spanning sequence) are assumed to be PCR or sequencing errors, and are discarded. Then, by counting the number of barcode sequences that are associated with a given rearrangement (clustering barcodes with a Levenshtein distance of 3 to allow for errors in the barcode sequence itself), we can estimate how many original cDNA molecules encoded the same TCR chain, reducing the impact of PCR duplication. Decombinator and error-correction scripts were written in Python. All remaining analyses were carried out in R, using the following packages: ineq (Gini index calculation), vegan (diversity indices), and igraph and RCytoscape (network/cluster analysis). IMGT TCR nomenclature and CDR3 definition (running from the last conserved cysteine in the V to the conserved phenylalanine in the FGXG motif in the J gene) have been used throughout (20). HIV-associated CDR3s were harvested from references (6, 21–38) and MAIT sequences from references (39, 40), while CMV-associated sequences were obtained via reference (41). Statistical significance was determined using *T* test (paired or unpaired as appropriate) except where indicated.

### 2.4 Data Availability

FASTQ data are available from the Sequence Read Archive, accession number SRP045430. Processed TCR data and published antigen-/subset-specific CDR3s are available for download (DOIs: 10.6084/m9.figshare.1153921 and 10.6084/m9.figshare.1153817). TCR analysis scripts are freely available (DOI: 10.6084/m9.figshare.1190861^1^), as is the standard version of Decombinator ((19), RRID:OMICS_00001^2^).

## 3 Results

Two unfractionated blood samples were taken from HIV^+^ patients – one immediately prior to (S1) and another 3 months after commencing ART (S2) – from which RNA was extracted. Ten uninfected donors were also sampled as controls, four of whom were sampled twice 3 months apart. RNA was reverse transcribed, and cDNA molecules were labeled with molecular barcodes before amplification and sequencing (Figure S1 in Supplementary Material).

Patient CD4 counts showed a modest but significant increase in most individuals (14/16) after 3 months of therapy, yet remained below normal values (Figure S2A in Supplementary Material). CD8 counts showed no consistent change over this period, while the CD4:CD8 T-cell ratio slightly increased (Figures S2B,C in Supplementary Material). Viral genome counts decreased rapidly and were undetectable in 11/16 individuals by the second sample collection (Figure S2D in Supplementary Material) and in all patients after a further year of ART. Clinical data and the number of sequences obtained are summarized in Table S2 in the Supplementary Material.

### 3.1 HIV^+^ TCR Repertoires Show a Profoundly Decreased Richness and Diversity Which Does Not Recover During Short-Term ART

**Figure 1:**
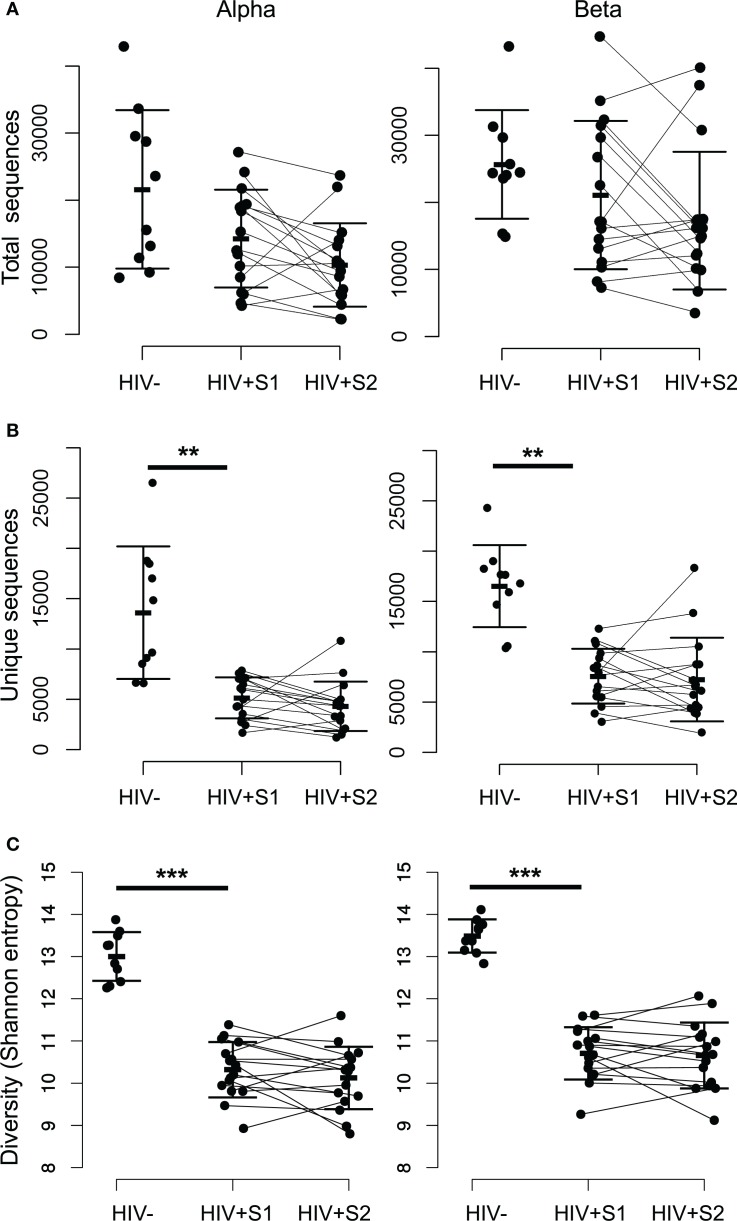
**The repertoires of HIV-infected individuals have a smaller number of distinct sequences, and lower diversity**. **(A)** The total number of alpha (left) and beta chain (right) CDR3 sequences obtained from each HIV^−^ and HIV^+^ individual, at both the pretreatment (S1) and treated (S2) bleed. The bars show mean and SD. **(B)** As for **(A)** but showing the number of unique CDR3 sequences observed within each set. The difference between the mean of the HIV^−^ and HIV^+^ S1 samples was significant, *p* < 0.01. There was no significant difference between HIV^+^ S1 and HIV^+^ S2. **(C)** As for **(A)** but showing CDR3 diversity measured by the Shannon information index (or Shannon entropy) for each sample.

The complementarity determining region 3 (CDR3) section of a given T-cell receptor is that which is encoded at the hypervariable recombination junction, and forms the primary contacts with the peptide-MHC antigen (42). Therefore, as the major site of biological interest, it is the primary sequence investigated in this study. There is no significant difference in the total number of TCR CDR3 sequences of either chain between HIV^−^ and HIV^+^ repertoires (Figure 1A), in agreement with the lack of total T-cell lymphocytopenia in these patients. In contrast, there is a significant decrease (*p* ≪ 0.01) in the number of distinct sequences observed (Figure 1B), as HIV^+^ samples contain on average threefold fewer sequences per chain. The TCR repertoire is determined both by the number of different sequences (richness) and the relative abundances of each clonotype. The combination of these properties is typically quantified as diversity, with decreased diversity likely reflecting a decreased ability of the repertoire to recognize and respond across the spectrum of potential pathogen antigens (43). One frequently used measure of diversity is the Shannon information index, or Shannon entropy, which is significantly decreased (*p* < 0.01) in untreated HIV^+^ repertoires (Figure 1C) and shows no recovery after therapy. This low diversity shows a weak inverse correlation to viral load (Pearson correlations: alpha chain R = −0.48, *p* = 0.06; beta chain R = −0.41, *p* = 0.12). Random sampling of the same number of sequences to produce size-matched repertoire files shows the same significant decrease in diversity, thus it is not a feature of the lower number of sequences found in patient repertoires (Figure S2E in Supplementary Material).

### 3.2 Altered V Gene Usage in Chronic HIV Infection

**Figure 2:**
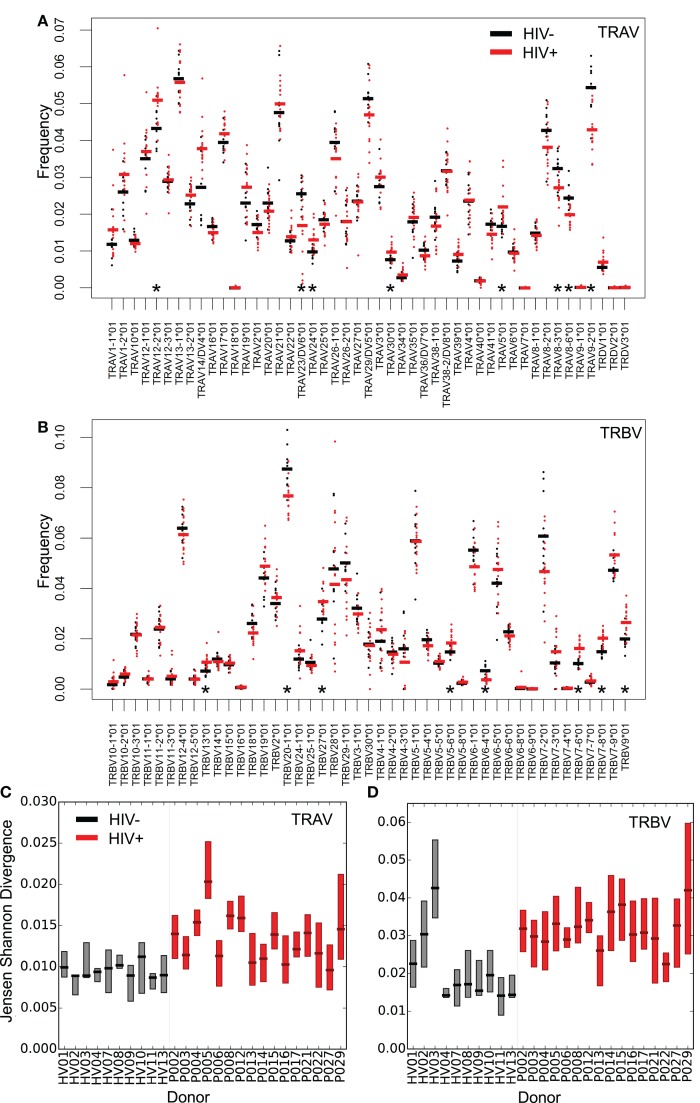
**Altered distribution and interindividual differences in V gene usage in the repertoires of HIV^+^ individuals**. The frequency of V alpha **(A)** and V beta **(B)** gene usage for the unique TCR repertoires of each donor sample within the two groups (HIV^−^ in black or untreated HIV^+^ in red). Bars show the mean proportion for each V gene in each group. Asterisks (*) show those V genes, which differ significantly in their usage (*T* test, *p* < 0.05) between HIV^−^ and HIV^+^. Interindividual differences in patterns of V gene usage (measured using Jensen–Shannon distances) for alpha **(C)** and beta **(D)** V genes. The plot shows the median and interquartile range of the Jensen–Shannon distances between each individual and all other individuals in turn from the same group. HIV^−^ are shown in black; HIV^+^ are shown in red.

The distribution of V gene usage for both chains, measured for distinct sequences (counting each TCR only once to avoid bias from the effects of clonal amplification), is shown in Figures 2A,B. As reported previously, the expression pattern of different V genes is non-uniform and conserved between individuals (44–46). Eight V alpha and eight V beta genes showed significant differences in mean expression between patients and controls, suggesting that HIV infection might differentially modulate certain parts of the repertoire, for example by driving expansion of HIV (or coinfecting pathogen) reactive T-cells using specific genes. A recent repertoire analysis of Treg cells sorted from HIV-infected individuals (47) showed a similarly non-uniform V and J gene distribution, with many of the most frequently used V genes observed in that study being similarly over represented in this study (e.g. TRBV6-5).

V gene usage profiles between HIV^+^ repertoires also differed by a greater amount than between healthy volunteer repertoires. This was measured by calculating Jensen–Shannon distances for each pair of V gene usage distributions; HIV^+^ pairs showed greater distances than HIV^−^ pairs (Figures 2C,D). Chronic HIV infection therefore leads to severe idiosyncratic perturbations of T-cell compartments, driving each individual’s repertoire away from typical ranges into their own distinct state. In contrast, the distributions of J gene differences showed few significant differences between HIV^+^ and HIV^−^ repertoires (Figure S3 in Supplementary Material). This may reflect the shorter length and greater propensity for base removal during recombination in the J genes relative to the V genes, and in the case of beta chain sequences, there being far fewer J genes for rearrangements to draw upon.

Comparing the distribution of V genes of HIV^+^ repertoires before and after therapy revealed that those relatively perturbed genes tended to return towards healthy values (Figure S4 in Supplementary Material) but changes observed were small.

### 3.3 Decreased TCR Sequence Sharing Between Repertoires of HIV^+^ Individuals

**Figure 3:**
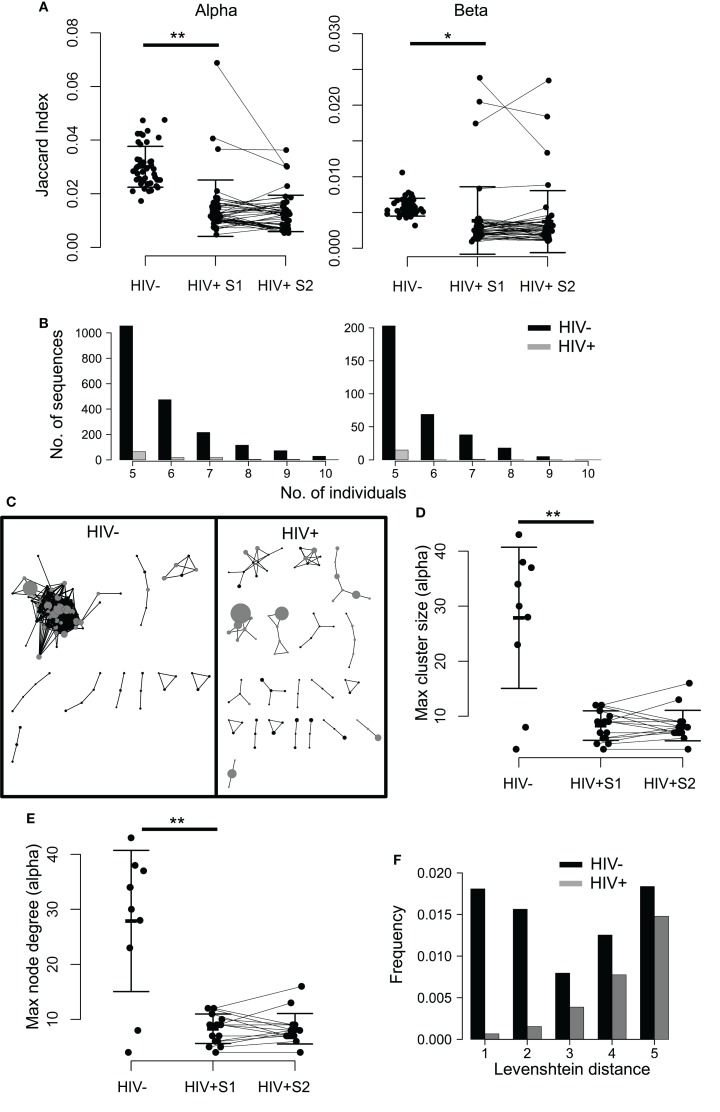
**TCR repertoires of HIV^+^ individuals show less sequence sharing and reduced TCR similarity networks**. **(A)** The proportion of shared sequences (the Jaccard index) between the repertoires of each pair of HIV^−^ and HIV^+^ individuals, at either time point (pre- or mid-treatment, S1 and S2, respectively). *T* test: **p < 0.01, *p < 0.05. **(B)** The number of CDR3s, which are shared between 5, 6, 7, 8, 9, or 10 out of ten HIV^−^ (black) or HIV^+^ (gray) individuals. In order to correct for the larger size of the HIV^+^ cohort (16 HIV^+^ versus 10 HIV^−^), the plot shows the average results of analyzing 100 random samples of 10 out of the 16 samples available. **(C)** A network plot showing clusters of related alpha chain CDR3s from a representative HIV^−^ or HIV^+^ repertoire. Each node represents a unique CDR3, and the diameter of the node represents the number of CDR3s in the repertoire. Two CDR3 nodes, which differ from each other by a Levenshtein distance of 1 are connected by an edge. The 100 most abundant CDR3s in each repertoire are shown in gray. Only clusters with three or more nodes are shown. The largest alpha chain cluster size **(D)** and the maximum degree **(E)** in each HIV^−^ or HIV^+^ repertoire before (S1) and after (S2) therapy. Bars show mean and SD. Means of HIV^−^ and HIV^+^ differ significantly (**p < 0.01). **(F)** The frequency distribution of CDR3s, which differ by a Levenshtein distance of 1–5 in HIV^+^ (black) or HIV^−^ (gray) repertoires.

Despite the stochasticity inherent in V(D)J recombination, TCR sequences can often be found to be shared between individuals, being classified as *private* when they occur in only a few individuals and *public* when found in many (45, 48–51). To see whether HIV infection impacts upon TCR sharing, we first compared the proportion of CDR3s shared between pairs of HIV^+^ individuals to that of HIV^−^ pairs by using the Jaccard index (a normalized measure of sharing, Figure 3A), which revealed a profound loss of sharing among HIV-infected patients relative to healthy controls. This property is not a feature of sample size, as size-matching data by random selection shows the same result (Figure S5A in Supplementary Material). Similarly, by counting the number of CDR3s that occur in 5 or more out of 10 HIV^−^ or HIV^+^ individuals, we see a decrease in the number of highly shared sequences (Figure 3B), again indicating a loss of TCR sharing between HIV^+^ patients and a tendency toward idiosyncratic repertoires. Figure S5B in Supplementary Material shows the intradonor Jaccard indices (i.e., the overlap of each individual’s S1 and S2 samples) for reference.

### 3.4 HIV Infection Is Associated with Disruption of Networks of Similar CDR3s

In order to explore the connectivity between CDR3s, we measured the distance between every pair of CDR3 amino acid sequences in an individual, using the Levenshtein distance (the minimum number of changes required to turn one sequence into another). We then constructed networks by creating an edge between unique CDR3 sequences (nodes), which differ by a distance of one (Figure 3C; Figure S6 in Supplementary Material). The size of the largest alpha CDR3 network (cluster size) and the maximum number of edges going to any one node (the maximal degree) for each individual are shown in Figures 3D,E. The maximum cluster size and the maximum network degree in the HIV^+^ repertoires are much smaller than the largest clusters in the uninfected repertoires, indicating landscapes of CDR3s that are less similar than those seen in controls. Healthy donor alpha repertoires are more similar, revealed by increased frequency of CDR3s, which differ by a Levenshtein distance of five or less (Figure 3F). No differences were seen between patients and controls for these parameters in the beta chain networks (Figure S7 in Supplementary Material), perhaps reflecting the greater diversity of the beta sequences owing to the presence of TRBD genes.

### 3.5 ART Is Accompanied by Large Changes in the Abundances of Individual Alpha and Beta Chain Sequences

**Figure 4:**
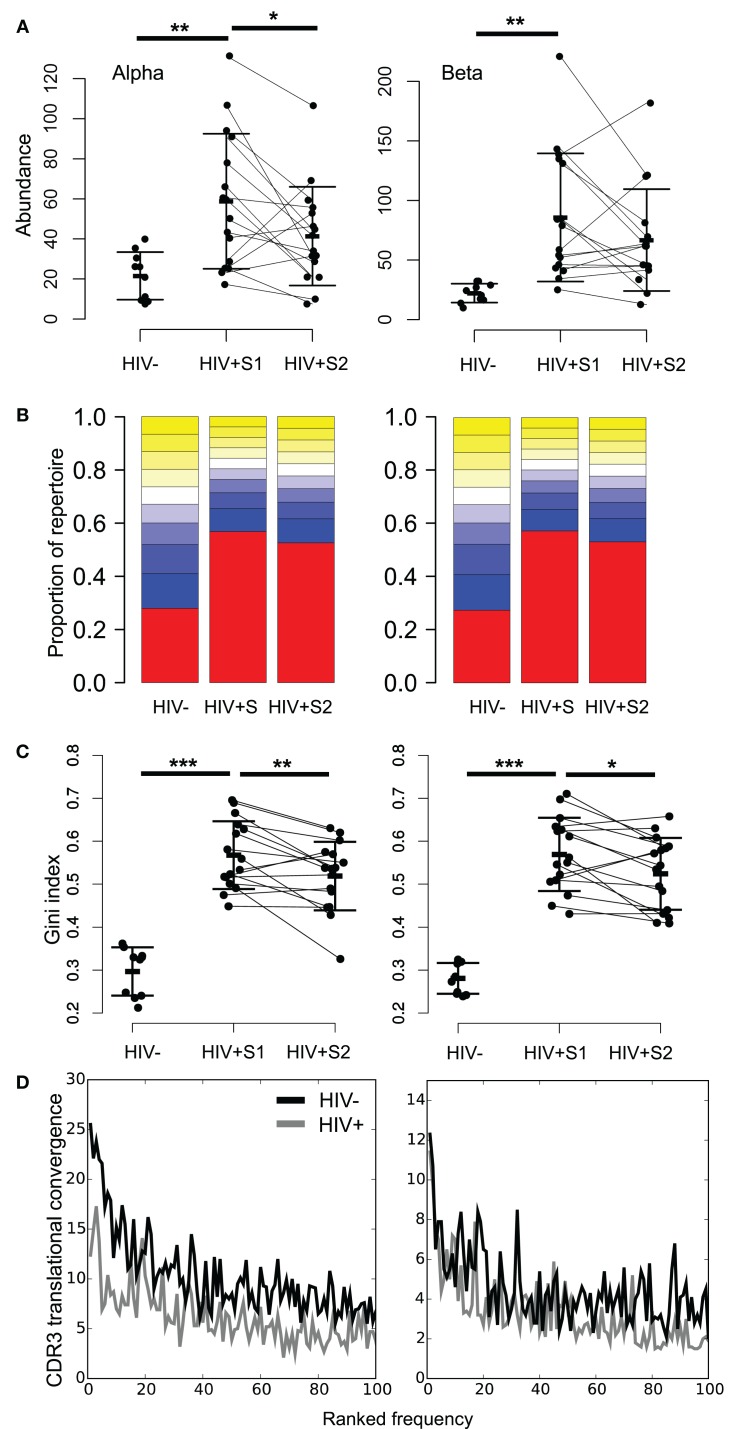
**HIV^+^ repertoires have a skewed TCR chain abundance distribution**. **(A)** The mean abundance of the 100 most abundant alpha (left) and beta chain (right) CDR3s in HIV^−^ and HIV^+^ before (S1) and after (S2) therapy. Bars show SD. *T* test: **p < 0.01, *p < 0.05. **(B)** Size distribution of TCR chain abundances. The plot shows the proportion of alpha or beta sequences in the HIV^−^ and HIV^+^ groups before (S1) and after (S2) therapy, which fall within each 10 percentile range of the size distribution. The percentile ranges are shown in decreasing order from the largest 10% (red) to the smallest 10% (yellow at top of each bar stack). **(C)** The Gini index of the TCR alpha or beta chain abundance distributions in HIV^−^ and HIV^+^ before (S1) and after (S2) therapy. *T* test: ***p < 0.001, **p < 0.01, and *p < 0.05. **(D)** Average translational convergence of alpha and beta CDR3s in HIV^+^ and HIV^−^ repertoires. The HIV^+^ and HIV^−^ distributions differ significantly (one-way ANOVA, *p* < 0.01).

By averaging the 100 most common sequences per donor, we observed that the most abundant CDR3s in HIV^+^ samples are significantly more frequent than controls (Figure 4A; Figure S8A in Supplementary Material). While the average frequency falls slightly following therapy, it remains higher and more variable than in HIV^−^ controls. These highly abundant sequences skew the repertoire frequency distributions of HIV^+^ individuals, with the most common sequences in patients occupying over twice as much of the repertoire than the most common sequences found in uninfected controls (Figure 4B).

The Gini index is a measure of unevenness that can measure this skewness, which ranges from zero for a uniform population (all TCRs being equally present) and tending toward one (maximum inequality). The mean Gini index is significantly increased in HIV^+^ samples compared to the healthy controls (Figure 4C), which could be considered reflective of increased oligoclonality. In contrast to the diversity as measured by Shannon entropy, the mean Gini index shows a significant recovery following therapy, yet remains above the mean of healthy controls. However, this observation is not replicated in size-matched data (Figure S8B in Supplementary Material). Interestingly, the Gini indices of the pre-treatment patient data significantly correlate to their CD8 counts (alpha R = 0.63, *p* = 0.01; beta R = 0.50, *p* = 0.05) but are independent of CD4 counts (alpha R = 0.21, *p* = 0.44; beta R = 0.06, *p* = 0.81). It is worth noting that the slight differences between the age and sex profiles of our control and uninfected donor cohorts fail to explain the differences observed in either measure of diversity (Figure S9 in Supplementary Material).

Because of the degeneracy of the genetic code and the similarity of germline TCR genes, identical CDR3s can be created by multiple DNA sequences (here referred to as translational convergence). By plotting the average number of different TCR rearrangements which translate to produce the 100 most abundant CDR3s in each group, we see lower translational convergence in the patient group (Figure 4D). Thus, despite the abundant CDR3s in HIV patients being far more frequent, they are translated from fewer DNA sequences. This suggests that abundant CDR3s in HIV patients arise from large clonal expansions.

**Figure 5:**
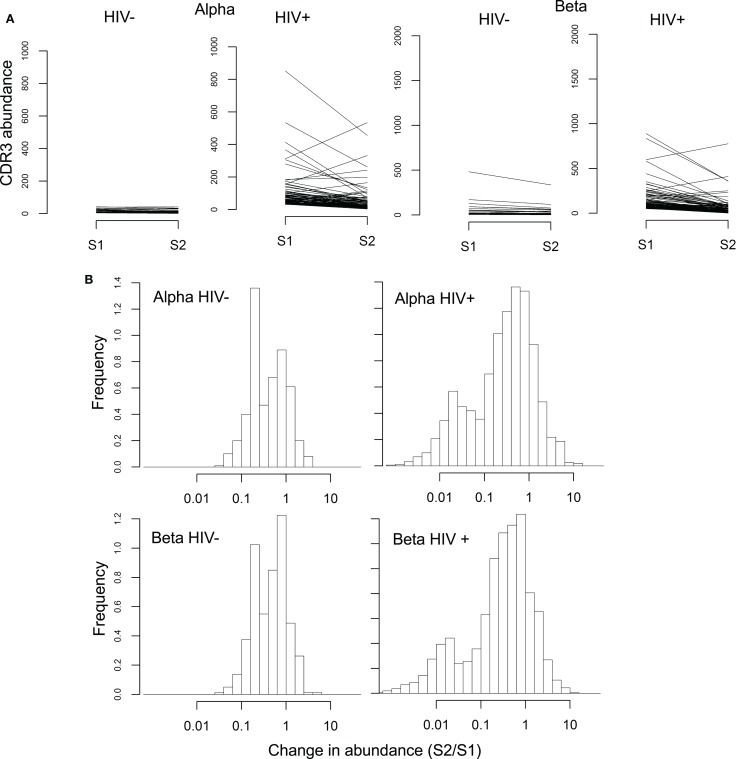
**Rapid changes in abundance of individual TCR sequences following ART**. **(A)** Abundance of the 100 most common alpha and beta sequences before (S1) and after (S2) ART therapy, in a representative HIV^−^ and HIV^+^ individual. **(B)** Change in abundance of the 100 most common alpha and beta sequences following ART, expressed as a ratio of abundance in S2 over the abundance in S1. Sequences absent in one of the two samples were assigned a size of 1 in that sample, so that the ratio between the two samples did not become infinite.

We tracked the frequencies of the 100 most abundant sequences per chain before and after therapy (example donors shown in Figure 5, with all donors in Figure S10 in Supplementary Material). Frequency histograms of the ratio of sequence abundance in S2 versus S1 (Figure 5B) show that HIV^+^ samples show more dispersion than controls, indicating that these sequences undergo larger frequency changes compared to the most common sequences in uninfected donors.

The histogram of the HIV^+^ samples shows one peak close to 1 (similar to HIV^−^ histograms) and another near 0.01, i.e., a decrease of approximately 100-fold. These changes occurred over 14 weeks which translates to a half-life on the order of 30 days, compatible with the known half-life of effector memory T-cells (52).

### 3.6 Mucosal-Associated Invariant T-Cell Sequences Are Depleted in HIV^+^ Repertoires and Do Not Recover Following ART

**Figure 6:**
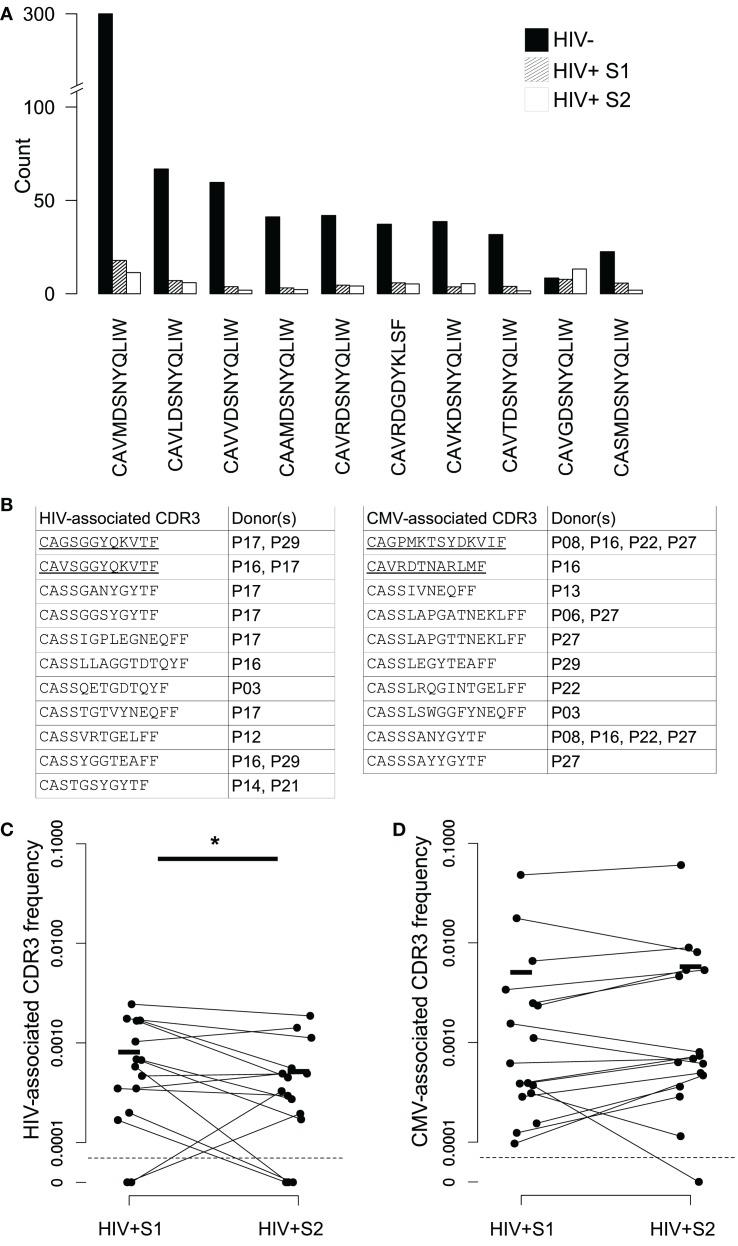
**Changes in frequency of MAIT and virally associated CDR3 sequences in HIV repertoires and following ART**. **(A)** Average numbers of the ten most frequent MAIT alpha chain sequences in the HIV^−^ (filled bars) and HIV^+^ repertoires before (dashed bars) and 3 months after (open bars) the start of ART. All sequences required an exact sequence match to a published MAIT CDR3 in the context of the correct germline V and J TCR genes. These genes are TRAV1-2 and TRAJ33, with the exception of CAVRDGDYKLSF, which instead uses the non-canonical TRAJ20 pairing. **(B)** Published CDR3 sequences from T-cells reported to be HIV or CMV specific were found in the repertoires of the HIV^+^ individuals shown. CDR3 sequences were only included if they occurred at an abundance of five or greater in either of the two of a patient’s samples. Underlining indicates alpha chain sequences, the rest being beta chain. **(C,D)** The proportion that each CDR3 listed in **(B)** occupies in the repertoires in which they were found, in both their pretreatment (S1) and midtreatment (S2) samples, for both HIV- and CMV-associated CDR3s (**(C,D)**, respectively). Horizontal lines indicate mean frequencies. The average clone size of the HIV CDR3s in sample 2 was significantly less than the size in S1 (*T* test, *p* < 0.05).

We searched our data for sequences belonging to non-MHC restricted T-cell subsets, many of which use TCRs which include “invariant” alpha chains, those which use a tightly conserved selection of V and J genes and near-identical CDR3s. Sequences associated with invariant natural killer T (iNKT) and germline-encoded mycolyl lipid-reactive (GEM) cells were extremely rare in our data, consistent with a reported very low frequency in blood. In contrast, CDR3s derived from mucosal-associated invariant T (MAIT) cells – which can constitute upwards of 10% of the healthy CD8^+^ T-cell pool (53) – were present in the context of the correct genes (TRAV1-2 with TRAJ33, TRAJ12, or TRAJ20) at high frequencies in healthy controls (Figure 6A). MAIT CDR3s were severely depleted in HIV^+^ repertoires and showed no evidence of recovery following therapy. V genes from beta chains known to pair with the invariant MAIT alpha chains (TRBV6-1 and TRBV20-1) (39) were among the few that were significantly decreased in the repertoires of HIV-infected donors (Figure 2A).

### 3.7 ART Induces Rapid Changes in the Numbers of HIV- and CMV-Associated CDR3s

While this study was not designed to gather alpha-beta chain pairing information, there are a number of reports in which even single chains from T-cells reactive against a particular antigen share sequence identity or features, and some TCRs can recognize the same peptide in the context of different MHC molecules (26, 54–56). Furthermore, there are several published reports of HIV-specific public TCR responses (21, 32, 33), sometimes even finding the same CDR3 in combination with different V and J genes (57). We therefore collected the CDR3 sequences of HIV-specific T-cells from the literature (92 alpha and 702 beta) and searched for matches in our data. A set of CDR3s from CMV-specific T-cells were also collected (13 alpha and 253 beta), as an example of another chronic virus known to drive large oligoclonal expansions (58–60). Because these sequences were typically obtained by isolating viral-specific pMHC-tetramer^+^ cells, the HLA restriction of the set was highly skewed to a small number of different genotypes.

We detected 15 examples of expanded HIV-associated CDR3s in our data (equal to or greater than five copies in at least one of a patients’ bleeds), comprised of 11 different CDR3s found across seven different individuals (Figure 6B). Some individuals had multiple matches, perhaps reflecting a match between their HLA type (not determined here) and those used to originally isolate the sequences. We found 17 examples of expanded CMV-associated CDR3s, comprised of ten different CDR3s observed in eight individuals. There was considerable overlap between the set of patients containing HIV- and CMV-associated CDR3s, again perhaps reflecting HLA genotype. Only two virally associated CDR3s were found in this analysis in the uninfected repertoires and only at the edge of detection at a single time point. As a control, we selected ten similarly sized sets of CDR3s from an independent published set derived from patients with Juvenile Idiopathic arthritis (61). Only an average of 4.5 of these CDR3s (±SD of 0.8) were found in our patients, suggesting the viral-associated CDR3 frequencies do indeed reflect actual virus exposure. Over the course of therapy, HIV-associated CDR3s significantly decreased in frequency, while CMV-associated sequences did not significantly alter (Figures 6C,D), although the majority of them (12 out of 17) did become more frequent.

## 4 Discussion

We have developed a high-throughput sequencing pipeline to measure quantitative parameters of the TCR repertoires of a cohort of HIV-infected patients, before and 3 months after the start of antiretroviral therapy. From a small unsorted blood sample, we are able to extract tens of thousands of TCR sequences per donor, providing the most comprehensive view of the global impact of HIV infection upon the TCR repertoire to date. Despite a relatively small cohort size, the results robustly identify multiple aspects of immune dysregulation and document some interesting changes that follow initiation of ART.

Dynamic changes in the TCR repertoire are a common feature of many viral infections. However, chronic HIV infection presents a number of special characteristics. During chronic HIV infection, the repertoire is severely restricted, averaging threefold fewer distinct sequences than uninfected controls. This depletion is consistent with the short life span of infected CD4^+^ T-cells (1, 62) and significant cell death in bystander uninfected cells early in disease progression (3). The shrinkage of the repertoire is opposed by an overexpansion of a small proportion of TCRs. The decrease in CD4^+^ counts and CD4:CD8 ratios combined with the positive correlation between Gini index and CD8^+^ T-cell count suggest that repertoire skewing can be attributed both to the decline in CD4 cells and large expansions, primarily in the relatively oligoclonal CD8 compartment. This hypothesis needs to be tested further by analyzing the TCR repertoire of sorted CD4 and CD8 T-cell populations, as well as various effector and memory subsets. It is also worth noting that large clonal expansions might not necessarily be directed against HIV antigens but might recognize those belonging to other common coinfections: indeed, there is some evidence that the majority of T-cell responses in untreated patients are not directed toward HIV peptides (63).

HIV infection was also associated with qualitative repertoire changes. Repertories from healthy adults are characterized by highly conserved non-uniform distributions of different V and J genes, thought to reflect intrinsic features of the recombination process (45, 49, 64). These distributions are significantly altered in the repertoires of the HIV-infected individuals, which may partially reflect dramatic depletion of some non-classical T-cell subsets, such as MAIT cells, which preferentially use certain V and J TCR genes. The HIV-associated decrease in MAIT cells has been reported previously (53, 65) and is consistent with the extensive damage to the gastrointestinal immune compartment, which is a feature of HIV infection (66, 67). Further studies looking at the repertoires of gamma-delta T-cells – which are enriched in gastrointestinal tissues (68) – and those of all T-cells from gut-associated lymphoid tissue will be interesting in this regard.

Healthy repertories are also characterized by the presence of public CDR3s, which are shared between many different individuals (45, 48–51). We also observed that repertoires from healthy donors contain sets of CDR3s with very similar amino acid sequences and that these sets often contain some of the most abundant CDR3s observed. Here, we observed that HIV infection associates with a profound depletion both of the number of CDR3s shared between people and CDR3 similarity within individuals. This decreased sharing may, in part, reflect differences in HLA types between individuals, although we have no reason to believe the HLA diversity is greater within the HIV cohort than among the controls. It may also be driven by heterogeneity in the microbial antigens presented over the course of infection, both in terms of the HIV mutating and in the complement of other infections a given patient has. The function of public CDR3s and CDR3 networks remain unknown, although both features have been suggested to play a role in the maintenance of self-tolerance (50). Disruption of these features may therefore aggravate the immune dysregulation, which is a feature of chronic HIV infection.

ART is a rapid and effective means to lower viral burden and initiate restoration of immune function. Paradoxically, recovery of the many parameters of immune function (e.g. CD4 count) occurs much slower, requiring years to return to normal ranges, likely related to low adult thymic output. TCR diversity and gene usage patterns are at best only partially restored after 3 months of ART. However, at an individual sequence level, initiation of therapy is accompanied by many rapid sequence expansions and contractions, including a reduction in the frequency of HIV-associated CDR3s. The T-cell receptor repertoire is therefore highly dynamic and undergoes substantial reshaping, which accompanies the rapid fall in viral load. Further functional studies will be needed to establish the causal relationship between the individual clonal dynamics and the increased ability to control opportunistic infections, which results from ART. It will also be instructive to obtain longer follow up samples from the HIV-infected cohort to document the long-term reconstitution of the immune repertoire. In particular, it will be interesting to see if the aberrant clonal expansions observed in the HIV repertoires remain and play any role in the chronic inflammatory phenotype, which seems to contribute to long-term morbidity in HIV-infected individuals, even when viral load is controlled.

This study only processed blood from sixteen HIV^+^ individuals, which constitutes a major limitation regarding the possible widespread applicability of our findings. Additionally, our treated S2 bleeds were taken after only approximately 3 months of antiretroviral therapy, which might be considered a relatively short time period to observe immune reconstitution in adult humans, given their reduced thymic output. However, we do observe a significant recovery in CD4 cell numbers over this period, in agreement with studies tracking CD4 cell numbers in larger cohorts of patients, which often show that the first 3 months of treatment produce the greatest absolute and relative CD4 T-cell recovery (69, 70). A related sampling limitation is that concerning the difference in number of different TCR sequences obtained from either HIV^+^ individuals or uninfected controls, which makes their analysis non-trivial. One can try to address this by randomly selecting equally sized samples from both groups, yet this results in analyses effectively including a higher proportion of rare events from the HIV^+^ data.

One of the major strengths of the approach described in this study is that all findings are derived from a fraction of RNA extracted from a small sample of peripheral blood. These samples are easily taken and stored, and using whole blood obviates the need to fractionate different cell populations, avoiding the cost, technical barriers, and health risks involved in processing potentially infectious biological material. However, this arrangement also imposes limitations on the depth of repertoire sequencing and the ability to separate out dynamics of different T-cell populations. If required – for instance to track rare T-cell clones – depth could be increased in future studies by taking more blood, processing more RNA or sequencing final libraries more deeply, yet as many of the interesting phenomena discussed here were manifest at a population structure level, this may not be required. Subpopulation information might also be inferred from correlations with routine clinical blood counts, as presented here, or might even be estimated from the properties of the TCR chains themselves, especially in the case of CD4 and CD8 T-cell sequence differentiation as their repertoires show marked divergence in several respects (71). This sequencing protocol could of course also be applied to sorted cell populations were such data required. Another limitation of the data presented here is one which affects the majority of the high-throughput TCR repertoire published data sets, thus far, in that we do not know which alpha chains pair with which betas in our samples. Such data would allow the determination of actual clonotypes and would permit cloning and functional testing of sequences of interest. However, while there are a number of protocols which potentially offer this ability – involving single-cell sorting (72), cellular emulsion PCR (73), in-cell nucleic acid linking (74), or bioinformatic inference of paired chains (75) – none are yet capable of producing data from the same volume of cells as bulk sequencing without large increases in handling time or cost.

This study therefore demonstrates a means by which TCR repertoire sequencing even from small, unfractionated blood samples – collection of which could be readily incorporated into a number of clinical studies – can be used to investigate a wide variety of immune parameters. Our results confirm and build upon a number of previous studies investigating repertoire perturbation during HIV infection. We are able to simultaneously track repertoire diversity, gene usage, and specific sequences in the data produced from a single experiment. These data highlight the dynamic nature of the repertoire, which is rapidly reshaped following the ART-induced fall in HIV load. These dynamic changes may lead to improved immune function despite a persistent contraction of the repertoire. In contrast, long-term damage to the repertoire may be much more difficult to repair and may contribute to long-term morbidity. Further studies, following larger cohorts of HIV^+^ individuals through the various phases of HIV infection and after long-term therapy, will help determine the clinical and immunological correlates of perturbation of the TCR repertoire. These data will help inform a rational approach to treatment optimization and may offer novel biomarkers for patient stratification. Moreover, this study demonstrates the potential of TCR repertoire sequencing for monitoring patients with infectious or immunodeficient diseases.

## Author Contributions

Study was conceived by MN, BC, and JH. Experimental protocols were designed by JH, TO, and BC. Initial sample processing (RNA extraction) was performed by EG and JR under MN. All other experimental work was carried out by JH. Analysis was performed by JH, KB, and BC. Manuscript was prepared by JH, NF, and BC with input from all authors.

## Conflict of Interest Statement

The authors declare that the research was conducted in the absence of any commercial or financial relationships that could be construed as a potential conflict of interest.

## Footnotes

^1^https://github.com/JamieHeather/tcr-analysis

^2^https://github.com/uclinfectionimmunity/Decombinator/
