# Supplementary material for: Dynamic Perturbations of the T-Cell Receptor Repertoire in Chronic HIV Infection and following Antiretroviral Therapy
Source: Front Immunol. 2016 Jan 11;6:644. doi: 10.3389/fimmu.2015.00644 (PMC4707277; doi:10.3389/fimmu.2015.00644)
Supplement: Supplementary file 1 [file table_1.docx]

| Primer name | Sequence |
| --- | --- |
| αRC2 | GAGTCTCTCAGCTGGTACACG |
| βRC2 | ACACAGCGACCTCGGGTGGGAA |
| SP2-6N | [Phos]NNNNNNAGATCGGAAGAGCACACGTCTGAACTCCAGTCAC[SpcC3] |
| SP1-6N-Ix-αRC1 | ACACTCTTTCCCTACACGACGCTCTTCCGATCTNNNNNNxxxxxxACGGCAGGGTCAGGGTTCTGGATAT |
| SP1-6N-Ix-βRC1.1 | ACACTCTTTCCCTACACGACGCTCTTCCGATCTNNNNNNxxxxxxGGTGGGAACACCTTGTTCAGGTCCTC |
| SP1-6N-Ix-βRC1.2 | ACACTCTTTCCCTACACGACGCTCTTCCGATCTNNNNNNxxxxxxGGTGGGAACACGTTTTTCAGGTCCTC |
| P5-SP1 | AATGATACGGCGACCACCGAGATCTACACTCTTTCCCTACACGACGCTCTTCC |
| P7-X-SP2 | CAAGCAGAAGACGGCATACGAGATAGTxxxxxxACTGGAGTTCAGACGTGTGCTCTTCCGATC |
| P5s | AATGATACGGCGACCACCGAGATC |
| P7 | CAAGCAGAAGACGGCATACGAGAT |

Table S1: Primer sequences used in our TCR amplification protocol.

'N' indicates any random nucleotide (A, C, T or G); random hexamers are used to barcode each cDNA molecule with a random 12-mer sequence which is used to track individual amplicon lineages in the analysis. 'xxxxxx' represents an indexing sequence, which allow us to pool multiple samples on each MiSeq run. SP2-6N ligation oligo was ordered with a 5' phosphate group (to allow ligation to 3' of cDNA molecules) and a 3' spacer C3 moiety (which blocks ligation, preventing oligomerisation).
