# Supplementary material for: Dynamic Perturbations of the T-Cell Receptor Repertoire in Chronic HIV Infection and following Antiretroviral Therapy
Source: Front Immunol. 2016 Jan 11;6:644. doi: 10.3389/fimmu.2015.00644 (PMC4707277; doi:10.3389/fimmu.2015.00644)
Supplement: Supplementary file 2 [file table_2.docx]

|  |  | Alpha DCR data | | Beta DCR data | | | Alpha CDR3 data | | | Beta CDR3 data | |
| --- | --- | --- | --- | --- | --- | --- | --- | --- | --- | --- | --- |
| Sample | Bleed | No. unique DCR IDs | Total no. DCR IDs | No. unique DCR IDs | Total no. DCR IDs | No. unique DCR IDs | | Total no. DCR IDs | No. unique DCR IDs | | Total no. DCR IDs |
| HV01 | S1 | 10,707 | 18,158 | 14,962 | 23,962 | 8,764 | | 15,572 | 14,521 | | 23,595 |
|  | S2 | 9,388 | 15,587 | 13,631 | 23,177 | 7,668 | | 13,207 | 13,246 | | 22,788 |
| HV02 | v1 | 7,647 | 9,770 | 17,989 | 24,744 | 6,459 | | 8,442 | 17,462 | | 24,380 |
|  | v2 | 10,414 | 14,308 | 13,627 | 18,224 | 8,839 | | 12,538 | 13,335 | | 18,017 |
| HV03 | v1 | 10,055 | 13,450 | 10,755 | 15,121 | 8,228 | | 11,388 | 10,476 | | 14,891 |
|  | v2 | 9,119 | 13,120 | 12,008 | 17,560 | 7,604 | | 11,286 | 11,705 | | 17,294 |
| HV04 | v1 | 7,716 | 10,802 | 10,549 | 15,561 | 6,468 | | 9,238 | 10,265 | | 15,321 |
|  | v2 | 7,134 | 10,904 | 9,648 | 15,742 | 5,958 | | 9,232 | 9,364 | | 15,471 |
| HV07 | v1 | 21,320 | 34,533 | 24,747 | 44,381 | 17,464 | | 29,554 | 24,018 | | 43,264 |
| HV08 | v1 | 30,614 | 50,254 | 17,925 | 24,369 | 24,612 | | 42,923 | 17,454 | | 24,053 |
| HV09 | v1 | 21,919 | 39,920 | 19,446 | 31,910 | 17,613 | | 33,625 | 18,799 | | 31,279 |
| HV10 | v1 | 19,903 | 33,973 | 16,232 | 26,170 | 16,199 | | 28,720 | 15,736 | | 25,706 |
| HV11 | v1 | 17,289 | 27,832 | 18,624 | 30,163 | 14,096 | | 23,580 | 17,991 | | 29,670 |
| HV13 | v1 | 11,293 | 15,603 | 17,187 | 24,978 | 9,323 | | 13,173 | 16,651 | | 24,476 |
| P002 | S1 | 7,017 | 14,411 | 6,269 | 11,341 | 5,821 | | 12,507 | 6,042 | | 11,090 |
|  | S2 | 5,059 | 9,779 | 6,868 | 12,319 | 4,251 | | 8,616 | 6,677 | | 12,102 |
| P003 | S1 | 7,577 | 20,738 | 11,328 | 31,827 | 6,314 | | 18,373 | 10,807 | | 31,451 |
|  | S2 | 5,525 | 12,378 | 7,325 | 16,313 | 4,625 | | 10,963 | 7,050 | | 16,153 |
| P004 | S1 | 5,025 | 9,705 | 8,142 | 17,363 | 4,147 | | 8,490 | 7,765 | | 17,102 |
|  | S2 | 12,594 | 25,056 | 18,738 | 38,034 | 10,362 | | 21,972 | 17,899 | | 37,469 |
| P005 | S1 | 2,004 | 5,012 | 3,140 | 8,373 | 1,651 | | 4,278 | 2,994 | | 8,210 |
|  | S2 | 3,451 | 7,812 | 4,610 | 10,183 | 2,834 | | 6,674 | 4,365 | | 9,923 |
| P006 | S1 | 5,905 | 22,051 | 8,848 | 32,902 | 4,832 | | 19,380 | 8,261 | | 32,330 |
|  | S2 | 5,089 | 14,913 | 6,258 | 16,916 | 4,234 | | 13,157 | 5,923 | | 16,623 |
| P008 | S1 | 8,257 | 21,006 | 11,033 | 27,156 | 6,901 | | 18,917 | 10,528 | | 26,754 |
|  | S2 | 3,875 | 6,840 | 6,737 | 12,638 | 3,247 | | 6,048 | 6,495 | | 12,357 |
| P012 | S1 | 3,176 | 7,134 | 5,711 | 13,472 | 2,672 | | 5,982 | 5,484 | | 13,130 |
|  | S2 | 7,539 | 17,087 | 8,954 | 17,941 | 6,240 | | 14,045 | 8,652 | | 17,519 |
| P013 | S1 | 3,520 | 7,257 | 8,610 | 22,973 | 2,962 | | 6,214 | 8,153 | | 22,541 |
|  | S2 | 1,786 | 2,713 | 3,930 | 6,850 | 1,495 | | 2,282 | 3,800 | | 6,735 |
| P014 | S1 | 8,934 | 25,037 | 12,610 | 35,694 | 7,348 | | 21,756 | 12,034 | | 35,159 |
|  | S2 | 5,459 | 12,315 | 14,083 | 40,487 | 4,596 | | 10,499 | 13,633 | | 40,104 |
| P015 | S1 | 2,889 | 5,420 | 3,963 | 7,396 | 2,405 | | 4,643 | 3,823 | | 7,298 |
|  | S2 | 1,433 | 2,637 | 2,026 | 3,526 | 1,170 | | 2,259 | 1,958 | | 3,485 |
| P016 | S1 | 8,387 | 27,839 | 9,595 | 30,262 | 6,945 | | 24,201 | 9,184 | | 29,683 |
|  | S2 | 2,441 | 6,548 | 4,503 | 14,861 | 2,077 | | 5,810 | 4,322 | | 14,649 |
| P017 | S1 | 4,214 | 13,463 | 5,653 | 17,368 | 3,456 | | 11,966 | 5,334 | | 17,147 |
|  | S2 | 2,255 | 5,061 | 4,046 | 10,276 | 1,876 | | 4,439 | 3,859 | | 10,144 |
| P021 | S1 | 8,379 | 32,396 | 10,072 | 45,247 | 6,852 | | 27,165 | 9,682 | | 44,719 |
|  | S2 | 8,882 | 26,862 | 10,762 | 31,186 | 7,410 | | 23,684 | 10,362 | | 30,790 |
| P022 | S1 | 7,133 | 17,557 | 7,797 | 16,314 | 5,976 | | 15,283 | 7,589 | | 16,123 |
|  | S2 | 5,645 | 11,049 | 8,882 | 17,639 | 4,675 | | 9,449 | 8,678 | | 17,436 |
| P027 | S1 | 5,052 | 11,649 | 4,632 | 10,422 | 4,216 | | 10,173 | 4,480 | | 10,311 |
|  | S2 | 3,957 | 12,176 | 4,811 | 15,089 | 3,289 | | 10,575 | 4,685 | | 14,947 |
| P029 | S1 | 9,219 | 23,132 | 6,638 | 14,689 | 7,641 | | 19,161 | 6,455 | | 14,520 |
|  | S2 | 5,911 | 18,078 | 5,842 | 16,388 | 4,881 | | 15,189 | 5,654 | | 16,183 |

Table S2: Raw clinical data for all samples used in this paper. HIV+ patients are assigned de-identified classifiers in the format P001, P002 et cetera. HV = healthy volunteer. DCR = Decombinator assigned identifier. S1 indicates pre-treatment samples, S2 indicates samples taken approximately three months later, corresponding to three months of ART for the HIV patients.
