# Supplementary material for: Dynamic Perturbations of the T-Cell Receptor Repertoire in Chronic HIV Infection and following Antiretroviral Therapy
Source: Front Immunol. 2016 Jan 11;6:644. doi: 10.3389/fimmu.2015.00644 (PMC4707277; doi:10.3389/fimmu.2015.00644)
Supplement: Supplementary file 3 [file datasheet_1.pdf]

Supplementary Fig. S1

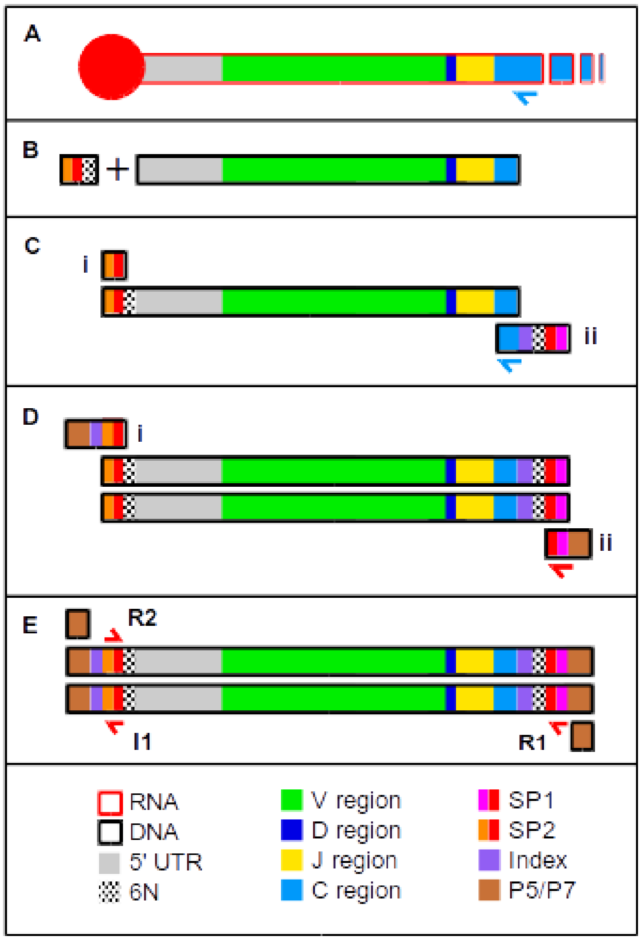

Fig. S1: **Schematic of TCR sequencing protocol.**

A: TCR RNA is reverse transcribed using oligonucleotides directed against the 5’ of the alpha and beta constant regions (blue arrow). Red circle represents mRNA 5’ cap.

B: RACE is achieved through ligation of DNA adapter to the 3’ end of the cDNA; adapter consists of Illumina sequencing primer SP2 and a hexamer of random nucleotides (6N).

C: Separate single round second (i) and third strand (ii) reactions allow incorporation of another random hexamer at the other end of the amplicons (as well as SP1, the other sequencing primer, and an index for demultiplexing), completing the unique 12-mer barcoding of TCR cDNA.

D: A four-cycle PCR is used to add: another index (which is sequenced in the dedicated Illumina indexing read) and P7 at one end (i) and P5 at the other (ii). P5 and P7 are the elements required for cluster generation, as they bind to the oligonucleotides that coat the flow-cell, permitting bridge amplification.

E: A final PCR directed against the P5 and P7 elements (for 23 cycles) amplifies full-length amplicons to sufficient concentrations for sequencing. Amplicons are purified, quantified, sized and normalised before sequencing on the MiSeq.

Supplementary Fig. S2

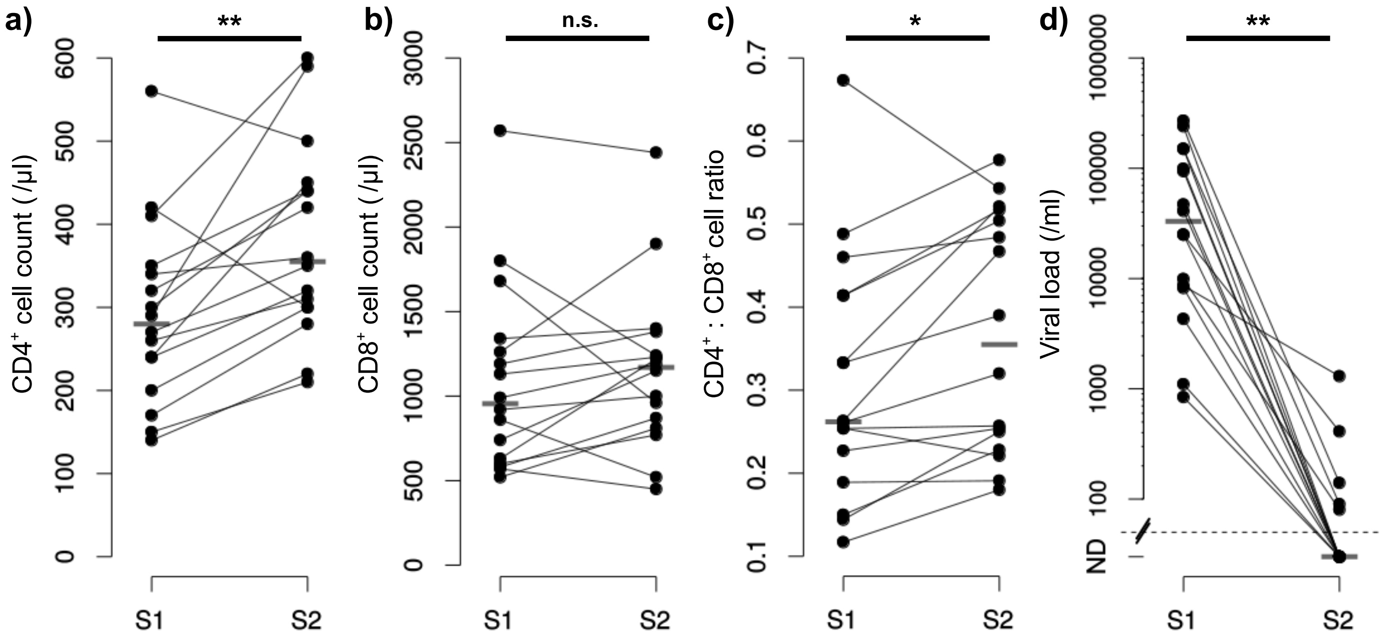

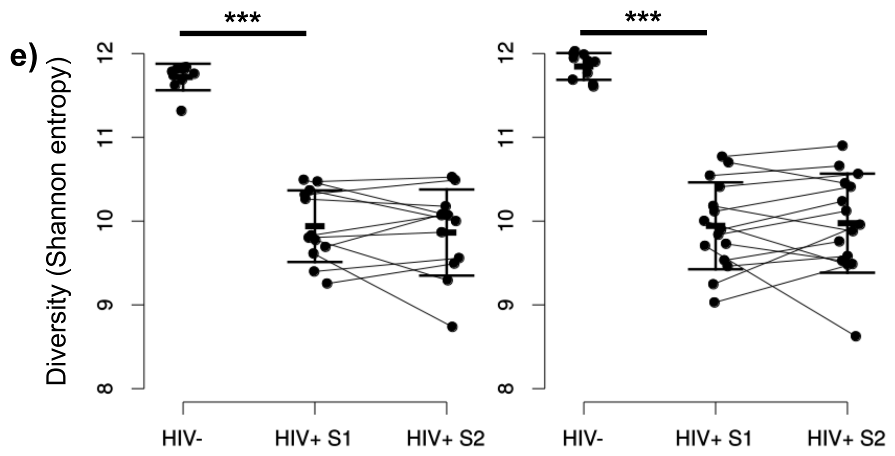

Fig. S2: **Patient clinical parameters and subsampled repertoire diversities.**

A-**D**: Changes in clinical parameters for the sixteen HIV+ patients both before (S1) and after three months of ART (S2), revealing a slight CD4 T-cell count recovery (**A**), no significant change in CD8 cell numbers (**B**), increase in CD4:CD8 ratio (**C**) and rapid fall in viral load (**D**). Each line represents one patient, and the horizontal line indicates the median. T test: * = p<.05, ** = p<.01).

E: Diversity as measured using Shannon entropy for size-matched samples produced by random selection of 5000 CDR3 sequences from whole repertoire samples to a fixed amount prior to diversity calculation. Samples with fewer than 5000 CDR3s were omitted. T test: *** = p<.001.

Supplementary Fig. S3

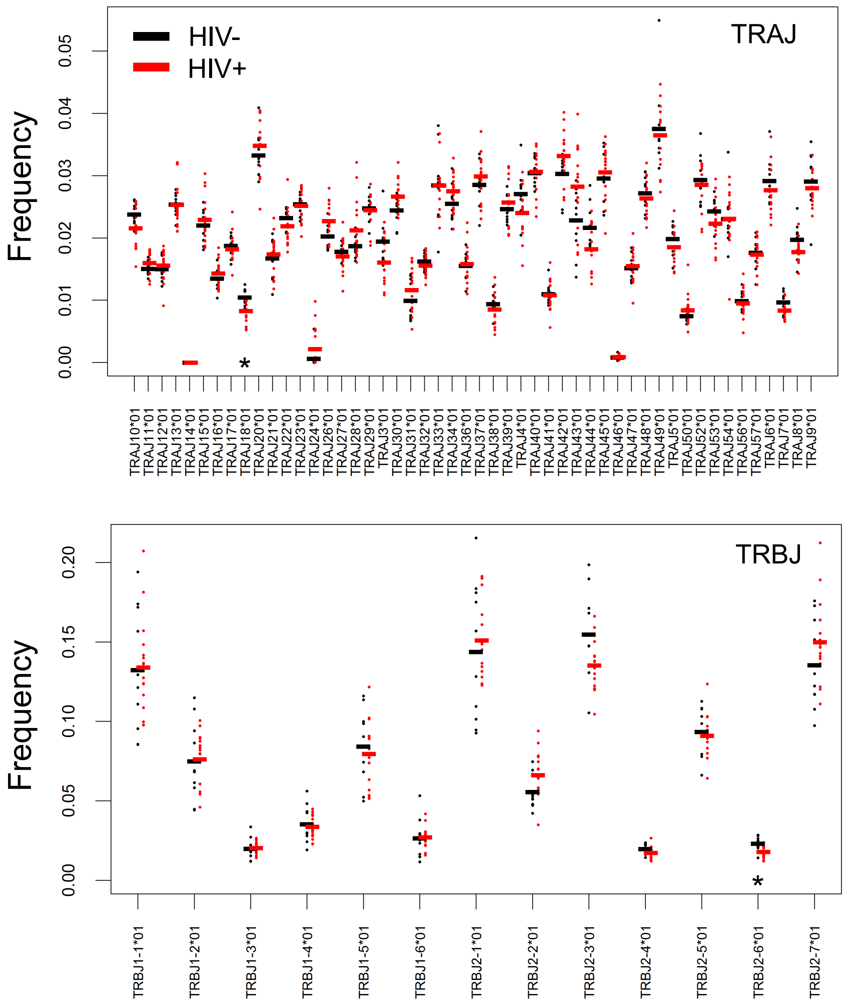

Fig. S3: **Altered distribution and heterogeneity of J gene usage in the repertoires of HIV infected individuals.**

The frequency of J gene usage for both alpha (top) and beta (bottom) chains within the set of unique TCRs for each HIV- (black circles) or HIV+ (red circles) sample. Bars show the mean proportion for each J gene. * show J genes which differ significantly (T test, $p < 0.05$) between HIV- and HIV+ repertoires.

Supplementary Fig. S4

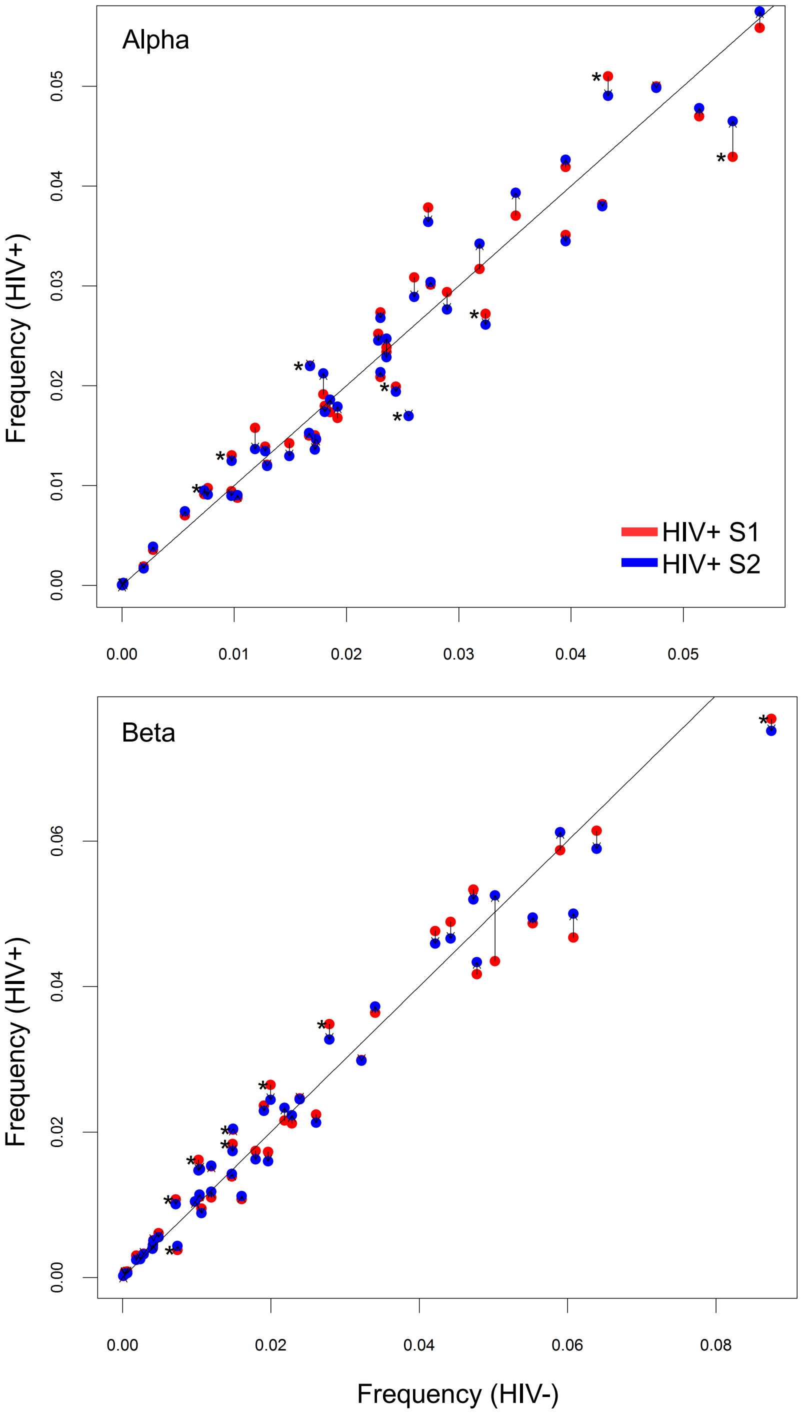

Fig. S4: **Changes in V gene usage after ART therapy**

The mean frequency of each V gene in HIV+ samples before (red) or after (blue) ART therapy is shown on the y axis plotted against the proportion found in HIV- samples (x axis), for alpha and beta chain repertoires (upper and lower panels respectively). Arrows show the direction of change after therapy. Asterisks show those V region which differ significantly between HIV- and pre-treament HIV+ samples.

Supplementary Fig. S5

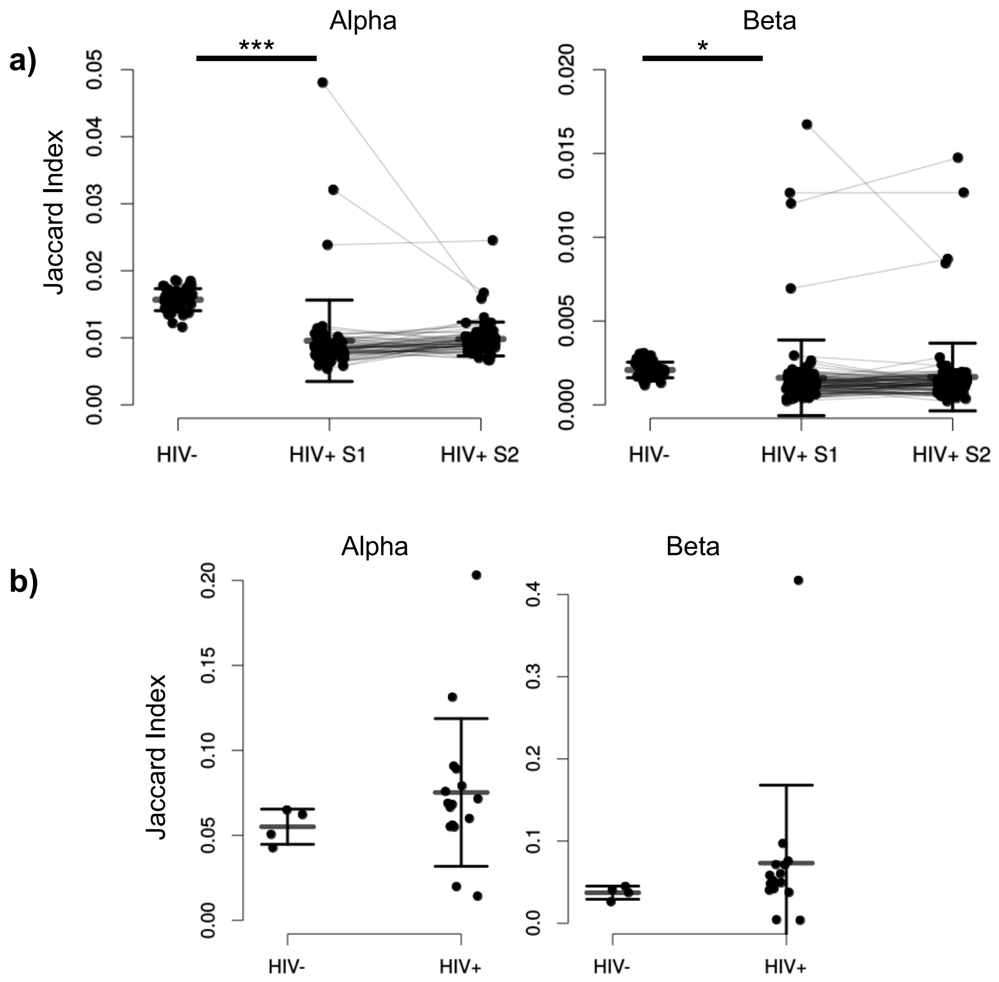

Fig. S5: **Loss of TCR sharing among HIV-patients is also seen in size-matched samples.**

A: The proportion of shared sequences (the Jaccard Index) between the CDR3 repertoires of each pair of HIV- and HIV+ individuals, at either time point (pre- or mid-treatment, S1 and S2 respectively), using data subsampled to 5000 CDR3s. T test: *** = $p<0.001$, * = $p<0.05$.

B: Whole (non-subsampled) repertoire intra-donor Jaccard indices (i.e. comparing the overlap between the S1 and S2 bleeds of the same individuals).

Supplementary Fig. S6

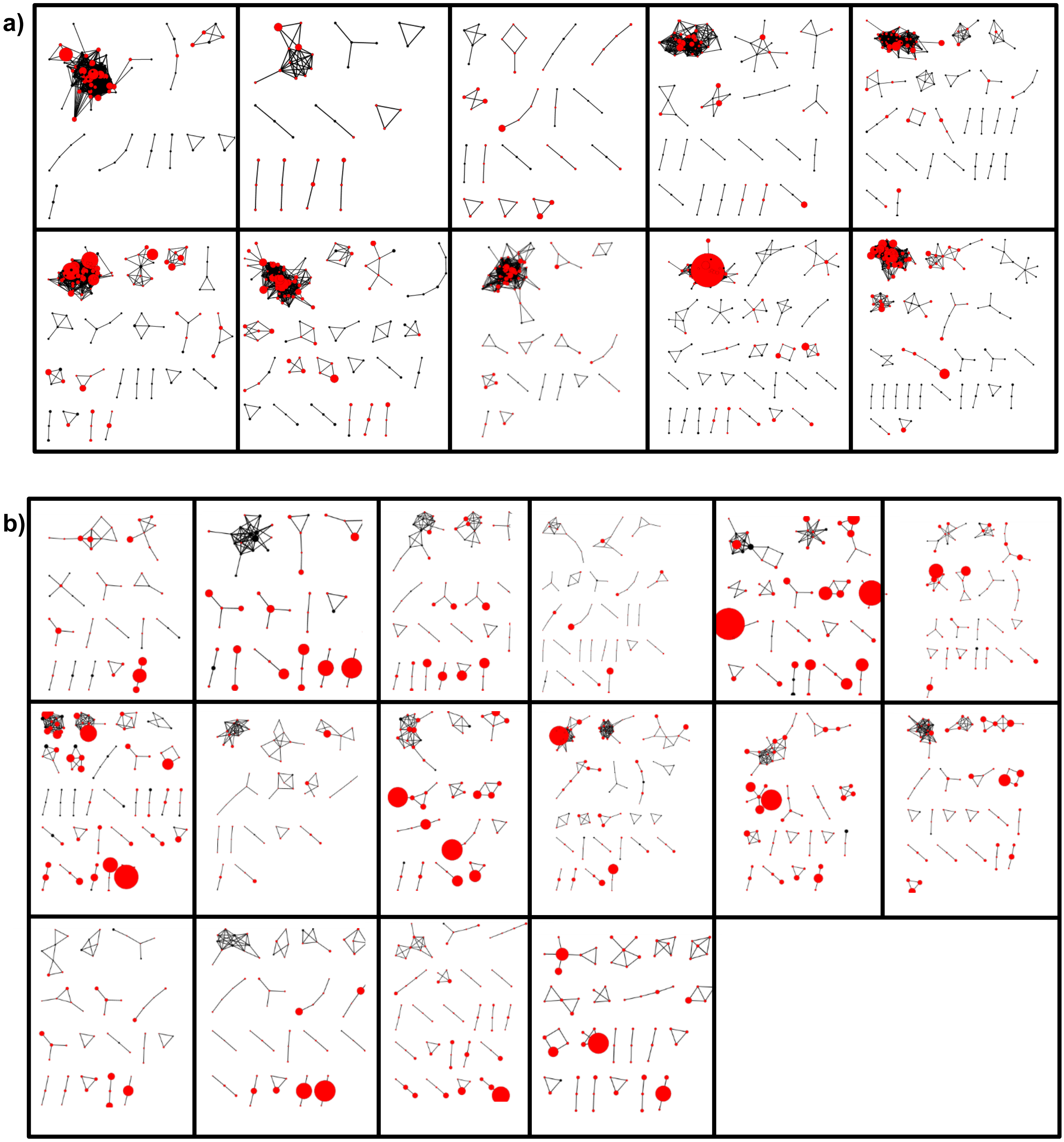

Fig. S6: **Cytoscape network plots showing clusters of related alpha chain CDR3s from all HIV- (A) or HIV+ (B) repertoires.**

Each node represents a unique CDR3, and the diameter of the node represents the number of CDR3s in the repertoire (clone size). Two CDR3 nodes which differ from each other by a Levenstein distance of 1 are connected by an edge. The 100 largest clones in each repertoire are shown in red. All clusters of size 3 or greater are shown.

Supplementary Fig. S7

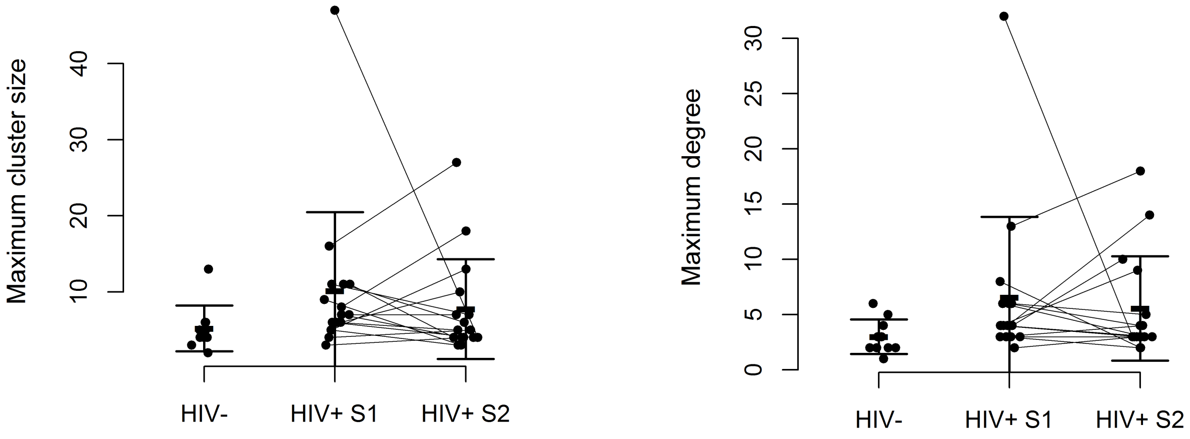

Fig. S7: **The largest beta chain cluster size and the maximum degree in each HIV- or HIV+ repertoire before (S1) and after (S2) therapy.**
Bars show mean and standard deviation.

Supplementary Fig. S8

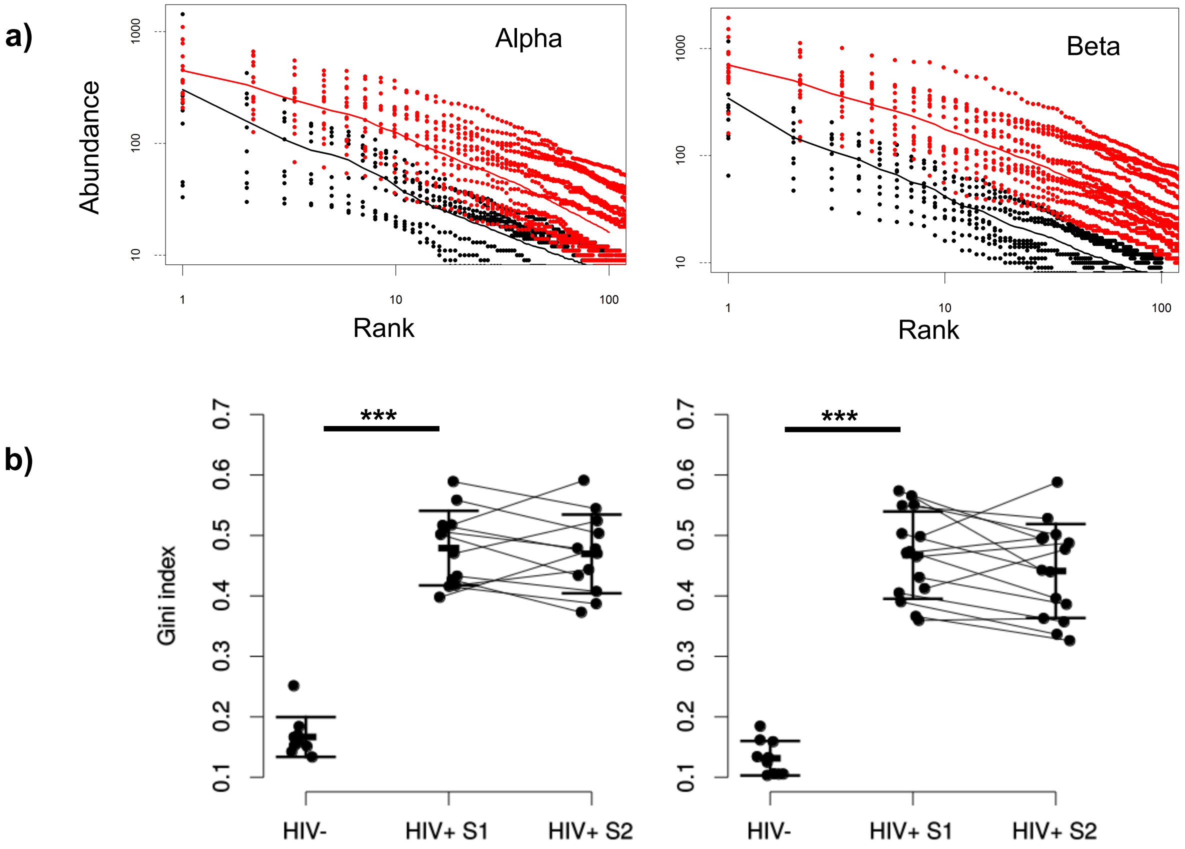

Fig. S8: TCR repertoires of HIV+ patients are dominated by very high-frequency sequences.

A: The size of the 100 largest sets of identical alpha chains (left) and beta chains (right) in each HIV+ (red) or HIV- (black) repertoire plotted in descending rank order. The mean for each set of repertoires is shown as a solid line.

B: The Gini indexes of each donor's CDR3 repertoire, after size-matching by sub-sampling to 5000 sequences, for alpha (left) and beta (right) chain samples. Patient repertoires show significantly higher scores (T test: *** = $p<0.001$), indicating repertoires with a relatively much more unequal distribution. Size-matched HIV+ samples fail to show the significant reduction in inequality on therapy as observed with whole samples (Fig. 4c).

Supplementary Fig. S9

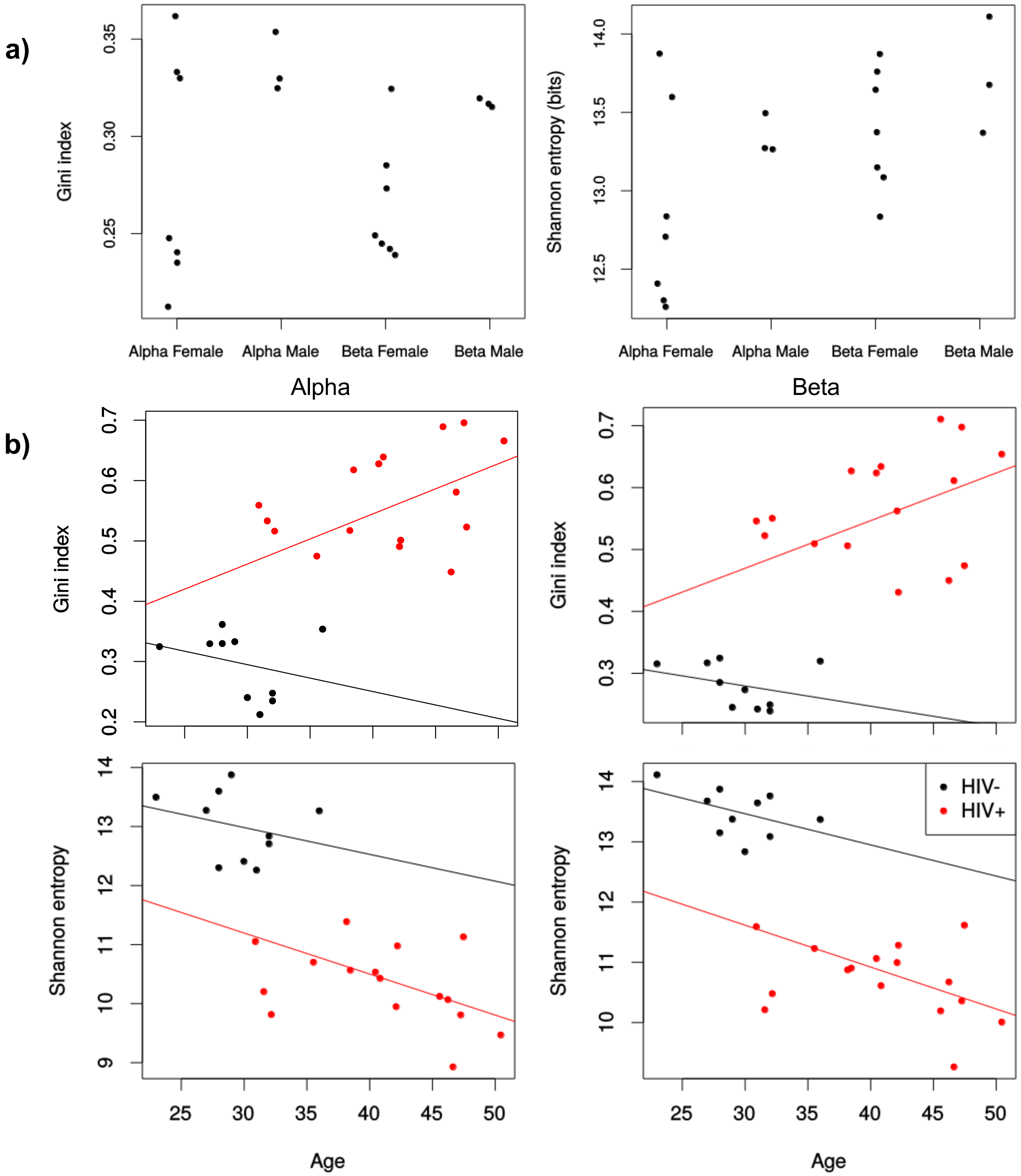

Fig. S9: Slight differences in the age and sex distributions between the control and infected cohorts do not explain the repertoire differences between the two populations.

A: Comparison of diversity metrics between males in females in the uninfected control cohort (there is only one female in the HIV+ group, hence this restriction). None of the distributions significantly differ between the sexes.

B: While age distributions differ between the populations, it is not the variable which drives the differences in diversity in either alpha (left) or beta (right) chain repertoires, as indicated by different distributions in the linear regressions of the two groups. Note that the only significant correlations are those between the age of HIV patients at time of sampling and their alpha diversity metrics ($p<0.05$).

Supplementary Fig. S10

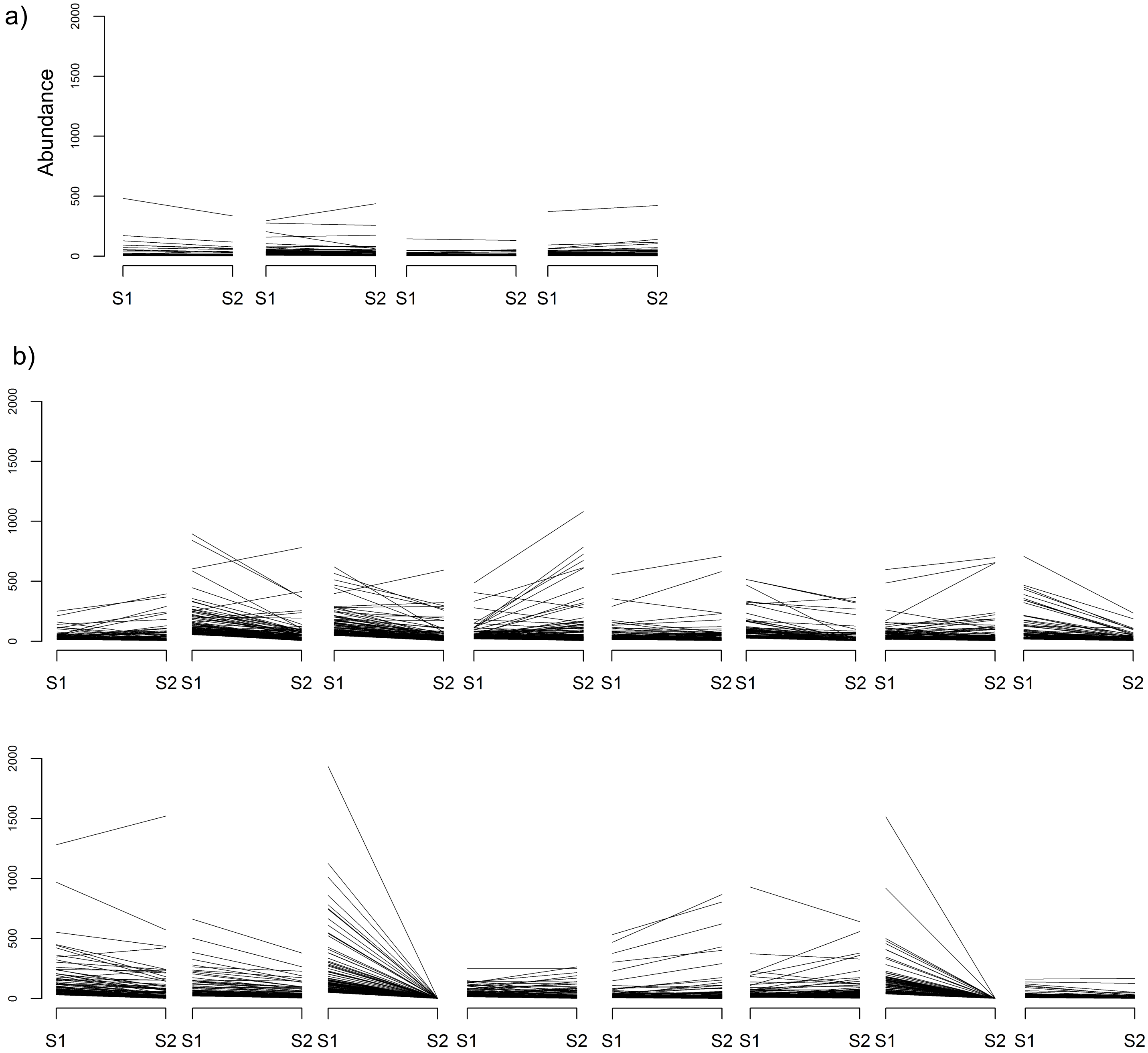

Fig. S9: **Rapid change in individual alpha chain abundances following ART.**

The abundance of the most abundant 100 alpha chain CDR3 sequences three months apart, in four HIV- **(A)** and 16 HIV+ individuals (**B**). S1 = first sample (pre-therapy in patients), S2 = second sample (post-ART).
